# Low let-7d and high miR-205 expression levels positively influence HNSCC patient outcome

**DOI:** 10.1186/s12929-019-0511-3

**Published:** 2019-02-13

**Authors:** Tomasz Kolenda, Kacper Guglas, Anna Teresiak, Renata Bliźniak, Katarzyna Lamperska

**Affiliations:** 1 Laboratory of Cancer Genetics, Greater Poland Cancer Centre, 15th Garbary Street, room 5025, 61-866 Poznan, Poland; 2 Department of Cancer Immunology, Chair of Medical Biotechnology, Poznan University of Medical Sciences, Poznan, Poland; 3 Postgraduate School of Molecular Medicine, Medical University of Warsaw, Warszawa, Poland

**Keywords:** Head and neck cancer, miRNA, Gene expression, Biomarker

## Abstract

**Introduction:**

Head and neck squamous carcinoma (HNSCC) is one of the most invasive types of cancer with high mortality. A previous study has indicated that low levels of let-7d and miR-205 in HNSCC patients are correlated with poor survival. Let-7d and miR-205 are tumor suppressors and regulators of epithelial-to-mesenchymal transition (EMT). However, it is unclear if let-7d and miR-205 together influence cancer cells.

**Aim:**

To determine if let-7d and miR-205 expression levels influence HNSCC patient outcome.

**Methods:**

The TCGA expression data for let-7d, miR-205 and their targets as well as clinical data were downloaded from cBioPortal and starBase v2.0 for 307 patients. The expression levels of let-7d and miR-205 were verified according to clinicopathological parameters. The let-7d and miR-205 high- and low-expression groups as well as disease-free survival (DFS), overall survival (OS) and expression levels of genes related to EMT, cancer stem cells, metastasis, cell cycle, drug response and irradiation response were investigated.

**Results:**

Let-7d and miR-205 were frequently upregulated in HNSCC compared to normal samples, and ROC analysis showed high discrimination ability for let-7d and miR-205 (area 0.7369 and 0.7739, respectively; *p* < 0.0001). Differences between expression levels of let-7d or miR-205 and grade, angiolymphatic invasion, perineural invasion and alcohol consumption were indicated. No differences were observed in N-stage, tumor localization, gender or patient age. Patients with lower let-7d levels and higher miR-205 levels had significantly better OS (*p* = 0.0325) than patients with higher let-7d levels and lower miR-205 levels. In the low let-7d level and high miR-205 level group, a lower percentage of more advanced cancers was observed. The analysis of genes related to EMT, cancer stem cells, metastasis, cell cycle, drug response and irradiation response revealed a distinct phenotype of analyzed groups.

**Conclusions:**

The present findings indicated that let-7d down-regulation and miR-205 overexpression create a unique cell phenotype with different behavior compared to cells with upregulated let-7d and down-regulated miR-205. Thus, let-7d and miR-205 are good candidates for new HNSCC biomarkers.

## Introduction

Head and neck squamous cell carcinoma (HNSCC) is characterized by high mortality and difficult to treat type of cancer. The main treatment of HNSCC involves surgical resection, radiotherapy and chemotherapy [1, 2]. Many reports have shown a close connection between HNSCC and miRNAs [3]. miRNAs are RNAs that are 22 nucleotides long and function as posttranscriptional regulators of specific mRNA by targeting the 3’ UTR of mRNA, resulting in reduced expression of the encoded proteins. These small RNAs regulate many biological processes, such as cell cycle, apoptosis, EMT, cancer-initiating cells, metastasis, drug response and irradiation response. The implication of miRNAs in HNSCC development and progression is well documented. miRNAs may function as tumor suppressors and/or oncogenes [3, 4], and they may be used as potential biomarkers in oncology [5, 6]. Childs et al. proposed that low expression levels of let-7d and miR-205 are prognostic markers of HNSCC [7], but other reports have not confirmed this finding.

Expression of lethal-7d (let-7d) is dysregulated in many types of cancer, such as HNSCC, breast cancer, kidney cancer, retinoblastoma, pancreatic cancer and prostate cancer [4, 8]. Let-7d plays a crucial role in cancer development, progression and metastasis, and it acts as a tumor suppressor miRNA through regulating the expression of many oncogenes [4]. However, recent studies have indicated that let-7d also acts as an oncogene [9, 10]. Let-7d regulates the response to irradiation and drug exposure through changes in cell phenotype via the EMT process or by changes in multidrug resistant genes [11–13].

MiR-205 is altered in many cancers, including breast cancer, melanoma, renal cancer, glioblastoma, lung cancer and HNSCC [6, 7, 14, 15]. MiR-205 may function as a tumor suppressor, and it has been described as an epithelial marker [16, 17] and a modulator of the EMT process [15]. In contrast, miR-205 functions as an oncogene in breast cancer, cervical cancer and nasopharyngeal carcinoma [18, 19].

The present study analyzed the expression of let-7d and miR-205 in HNSCC patients based on TCGA data. The aim of the present study was to investigate the influence of let-7d and miR-205 expression levels on HNSCC patient outcome.

## Methods

### TCGA data

TCGA expression data of let-7d, miR-205 and selected genes (cell cycle, apoptosis, EMT, cancer-initiating cells, metastasis, irradiation response and drug response) as well as clinical data were downloaded from cBioPortal (head and neck squamous cell carcinoma, TCGA, Provisional, 530 sample data set) and starBase v2.0 for 307 patients and presented as miRNAseq RPM (Reads Per Million) and mRNA expression z-Scores (RNA Seq V2 RSEM; z = 2, RNA-Seq by Expectation Maximization) [20, 21]. All data is available online with common access.

### Data analysis

The expression levels of let-7d and miR-205 were analyzed in all HNSCC sample localizations depending on the following clinicopathological parameters: age (< 60 vs. > 60), gender (women vs. men), T-stage (T1 + T2 vs. T3 + T4), N-stage (N0 vs. N1 + N2 + N3), cancer grade (G1 + G2 vs. G3 + G4), cancer stage (I + II vs. III + IV), HPV p16 marker (negative vs. positive), perineural invasion (negative vs. positive), angiolymphatic invasion (negative vs. positive), disease surgical margin status (negative vs. positive) and lymphoid neck dissection status (negative vs. positive). The group of 307 patients was divided into high and low expression subgroups for let-7d and/or miR-205. The following groups were created based on cut-off values for miRNA expression of healthy samples: i) high let-7d + low miR-205 (*N* = 24 cases); ii) high let-7d + high miR-205 (*N* = 217 cases); iii) low let-7d + low miR-205 (*N* = 12 cases) and iv) low let-7d + high miR-205 (*N* = 54 cases). Disease free-survival (DFS) and overall survival (OS) were analyzed in the four subgroups. The selected clinicopathological parameters (cancer grade, cancer stage, T-stage, perineural invasion, angiolymphatic invasion and disease surgical margin status) were compared between the low let-7d level and high miR-205 level group and the high let-7d level and low miR-205 level group. The expression levels of selected genes related to cell cycle, apoptosis, EMT, cancer-initiating cells, metastasis, drug response and irradiation response were analyzed. Let-7d and miR-205 predicted targets were verified using the miRDB database [22] and target genes were compared between patients with the low let-7d level and high miR-205 level group and the high let-7d level and low miR-205 level group.

### Statistical analysis

All statistical analyses were performed by Graphpad Prism 5 (GraphPad Software San Diego, CA, USA) using the Shapiro-Wilk normality test, T-test, Mann–Whitney U test or one-way ANOVA with Dunn’s multiple comparison test. For DSF and OS analyses, the log-rank (Mantel-Cox) and Gehan-Breslow-Wilcoxon tests were used. In all analyses, a *p*-value < 0.05 was considered as significant. The heat map and clustering were generated using MORPHEUS, an online versatile matrix visualization and analysis software tool (https://software.broadinstitute.org/morpheus/).

## Results

### Let-7d and miR-205 are frequently upregulated in HNSCC

**Fig. 1:**
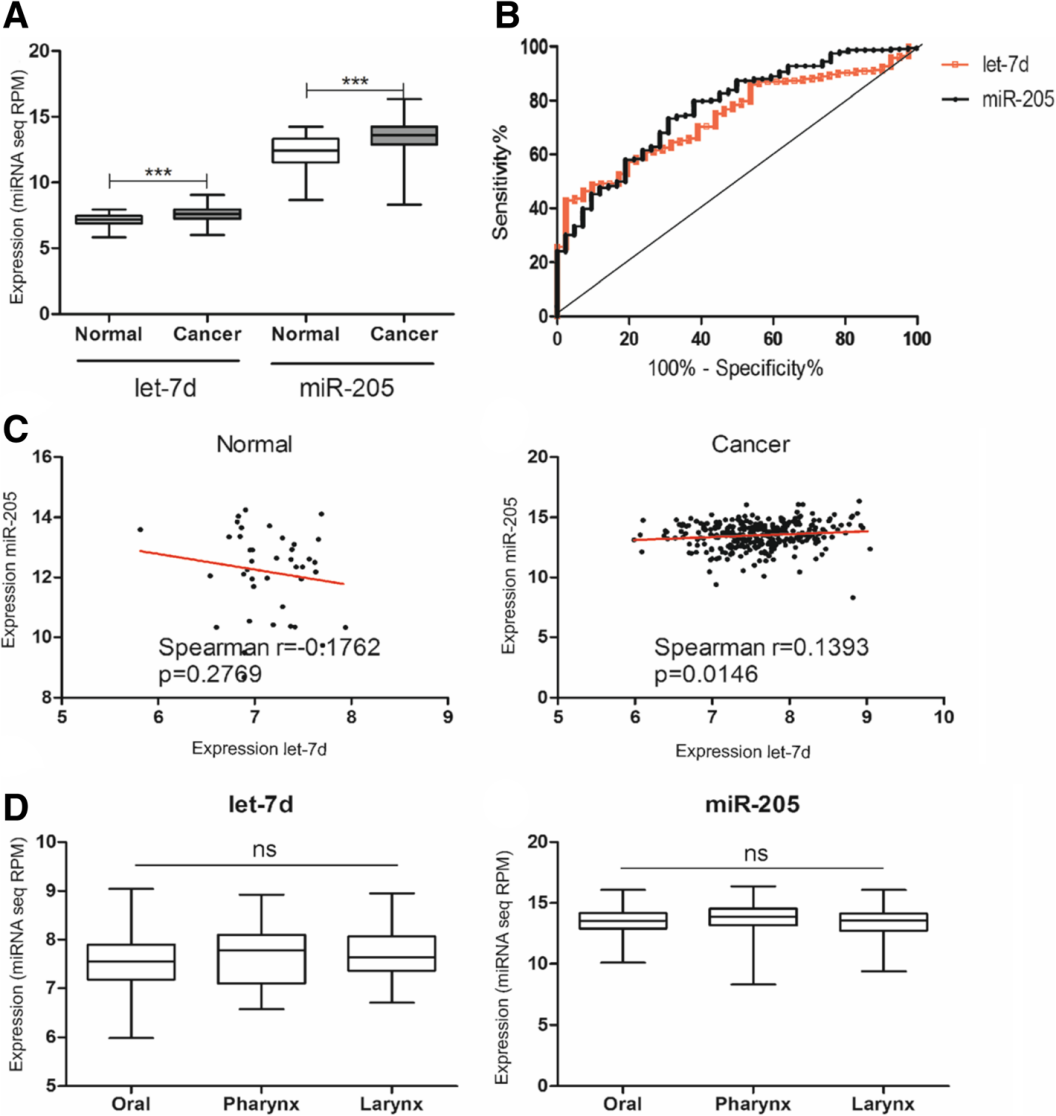
Characteristics of let-7d and miR-205 expression in normal and HNSCC tissues. **a**) Expression levels of let-7d and miR-205 in normal and cancer tissues; **b**) ROC analysis of let-7d and miR-205 expression; **c**) Spearman correlation of let-7d and miR-205 in normal and cancer tissues; **d**) Expression of let-7d and miR-205 depending on the tumor localization site. Mean of miRNAseq RPM expression with SEM; T-test or One-way ANOVA with Dunn’s Multiple Comparison Test; *p* < 0.05 was considered significant; ns – no significant, *** *p* < 0.001

Expression levels of let-7d and miR-205 in normal (*N* = 41 and *N* = 42 cases, respectively) and HNSCC tissues (*N* = 307 cases) were analyzed based on TCGA data (starBase v2.0). Both let-7d and miR-205 were frequently upregulated in HNSCC compared to normal samples (let-7d: 7.594 ± 0.03263 vs. 7.157 ± 0.06295, *p* < 0.0001; and miR-205: 13.50 ± 0.06195 vs. 12.21 ± 0.2130, *p* < 0.0001) (Fig. 1a). ROC analysis showed high discrimination ability of let-7d (AUC = 0.7369) and miR-205 (AUC = 0.7739) (both *p* < 0.0001) to discriminate cancer and healthy tissues (Fig. 1b).

The correlations between let-7d and miR-205 were also investigated. For normal samples, Spearman correlation analysis indicated no association between the let-7d and miR-205 miRNAs (R = − 0.1762, *p* = 0.2769). For cancer tissues, however, a positive correlation between let-7d and miR-205 expression levels was observed (R = 0.1393, *p* = 0.0146) (Fig. 1c).

The expression levels of let-7d and miR-205 were examined based on the oral (*N* = 193), pharynx (*N* = 39) and larynx (*N* = 75 cases) tumor localizations. No significant differences between localization and expression of both let-7d (*p* = 0.1305) and miR-205 (*p* = 0.1126) were identified (Fig. 1d).

### Expression level of let-7d and miR-205 reflecting clinicopathological parameters of HNSCC patients

The expression levels of let-7d and miR-205 were analyzed depending on clinicopathological context. Significant differences between expression levels of let-7d or miR-205 were observed with regard to grade (*p* = 0.0002 for let-7d; *p* = 0.5185 for miR-205), angiolymphatic invasions (*p* = 0.0024 for let-7d; *p* = 0.008 for miR-205), perineural invasions (*p* = 0.5565 for let-7d; *p* = 0.0165 for miR-205), alcohol consumption (*p* = 0.0227 for let-7d; *p* = 0.5678 for miR-205) and neck lymph node dissection (*p* = 0.1731 for let-7d; *p* = 0.0091 for miR-205). There were no statistically significant differences in the expression levels with regard to gender (*p* = 0.2445 for let-7d; *p* = 0.6210 for miR-205), patient age (*p* = 0.3846 for let-7d; *p* = 0.8371 for miR-205), N-stage (*p* = 0.0729 for let-7d; *p* = 0.3485 for miR-205), disease surgical margin status (*p* = 0.5931 for let-7d; *p* = 0.1907 for miR-205), HPV presence (*p* = 0.2600 for let-7d; *p* = 0.1429 for miR-205), cancer stage (*p* = 0.6672 for let-7d; *p* = 0.3658 for miR-205) and T-stage (*p* = 0.4987 for let-7d; *p* = 0.5219 for miR-205). All data is summarized in Table 1.

**Table 1:** Expression levels of let-7d and miR-205 depending on the clinicopathological parameters in HNSCC patients; T-test, *p* < 0.05 was considered significant

Parameter	Group	Cases analyzed	let-7d		miR-205	
			Mean ± SEM	*p*-val	Mean ± SEM	*p*-val
Age	< 61.3	159	7.621 ± 0.04545	0.3846	13.49 ± 0.08659	0.8371
	> 61.3	147	7.564 ± 0.04723		13.51 ± 0.08951	
Gender	Female	95	7.537 ± 0.06120	0.2445	13.59 ± 0.09850	0.621
	Male	221	7.619 ± 0.03845		13.46 ± 0.07811	
Alcohol	Yes	206	7.650 ± 0.03906	0.0227	13.5 ± 0.07550	0.5678
	No	97	7.491 ± 0.05784		13.5 ± 0.1092	
Grade	G1 + G2	219	7.515 ± 0.03841	0.0002	13.48 ± 0.06774	0.5185
	G3 + G4	80	7.796 ± 0.06118		13.57 ± 0.1461	
Cancer Stage	I + II	74	7.554 ± 0.06325	0.6672	13.52 ± 0.1002	0.3658
	III + IV	197	7.587 ± 0.04059		13.39 ± 0.08370	
T Stage	T1 + T2	118	7.555 ± 0.05406	0.4987	13.42 ± 0.08618	0.5219
	T3 + T4	154	7.602 ± 0.04398		13.44 ± 0.09825	
N Stage	N0	154	7.531 ± 0.04499	0.0729	13.55 ± 0.07323	0.3485
	N1 + N2 + N3	140	7.651 ± 0.04992		13.41 ± 0.1054	
Perineural Invasion	Yes	105	7.555 ± 0.05409	0.5565	13.27 ± 0.1050	0.0165
	No	110	7.601 ± 0.05588		13.62 ± 0.1045	
Angiolymphatic Invasion	Yes	67	7.762 ± 0.05671	0.0024	13.15 ± 0.1663	0.008
	No	139	7.506 ± 0.05074		13.59 ± 0.07981	
Disease Surgical Margin Status	Negative	210	7.550 ± 0.03765	0.5931	13.45 ± 0.07263	0.1907
	Positive	30	7.607 ± 0.09610		13.23 ± 0.1742	
Lymph Node Neck Dissection Indicator	Yes	246	7.575 ± 0.03530	0.1731	13.43 ± 0.07065	0.0091
	No	59	7.688 ± 0.08177		13.79 ± 0.1249	
HPV p16 status	Negative	16	7.730 ± 0.1301	0.26	13.63 ± 0.4052	0.1429
	Positive	48	7.552 ± 0.07919		13.32 ± 0.1816	

### Expression levels of let-7d and miR-205 influence patient survival

**Fig. 2:**
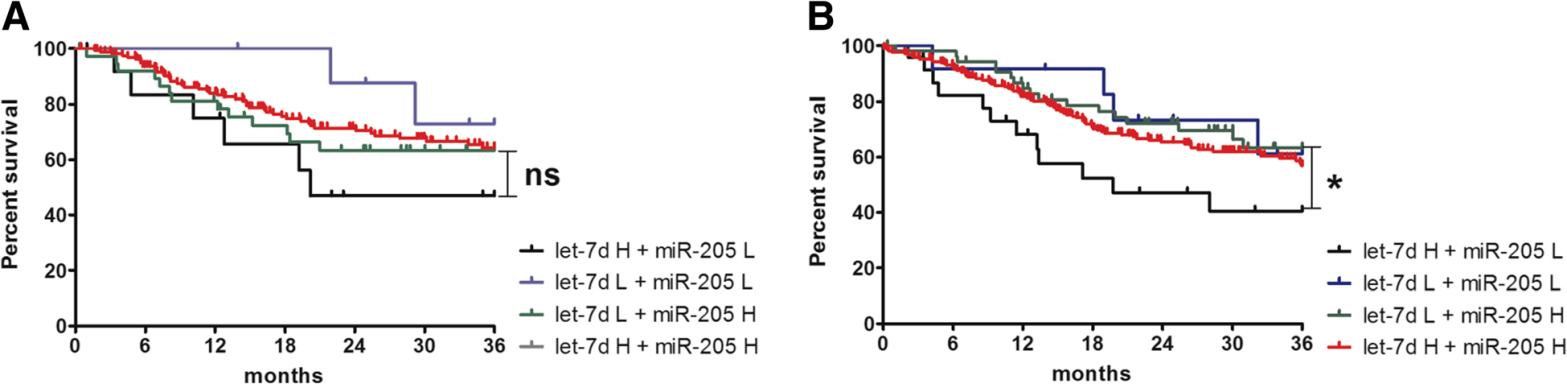
Disease-free survival (**a**) and overall survival (**b**) of HNSCC patients analyzed in groups of low/high expression of let-7d and miR-205. Groups were described in the material and methods section; *p* < 0.05 was considered significant, ns – no significant, * *p* < 0.05

The disease-free survival (DFS) and overall survival (OS) of HNSCC patients defined after 36 months of observation were analyzed in groups of different expression levels of let-7d and miR-205. There were no statistically significant differences between groups of patients with specified low and/or high expression levels of let-7d and miR-205. However, patients with low let-7d levels and high miR-205 levels had significantly better OS than patients with opposite phenotype - high let-7d levels and low miR-205 levels (*p* = 0.0325). These results are shown in Fig. 2a and b. Due to differences in survival between these groups, it was checked whether they differ in terms of other clinical features.

### Patients with low let-7d and high miR-205 expression levels have better prognosis

**Fig. 3:**
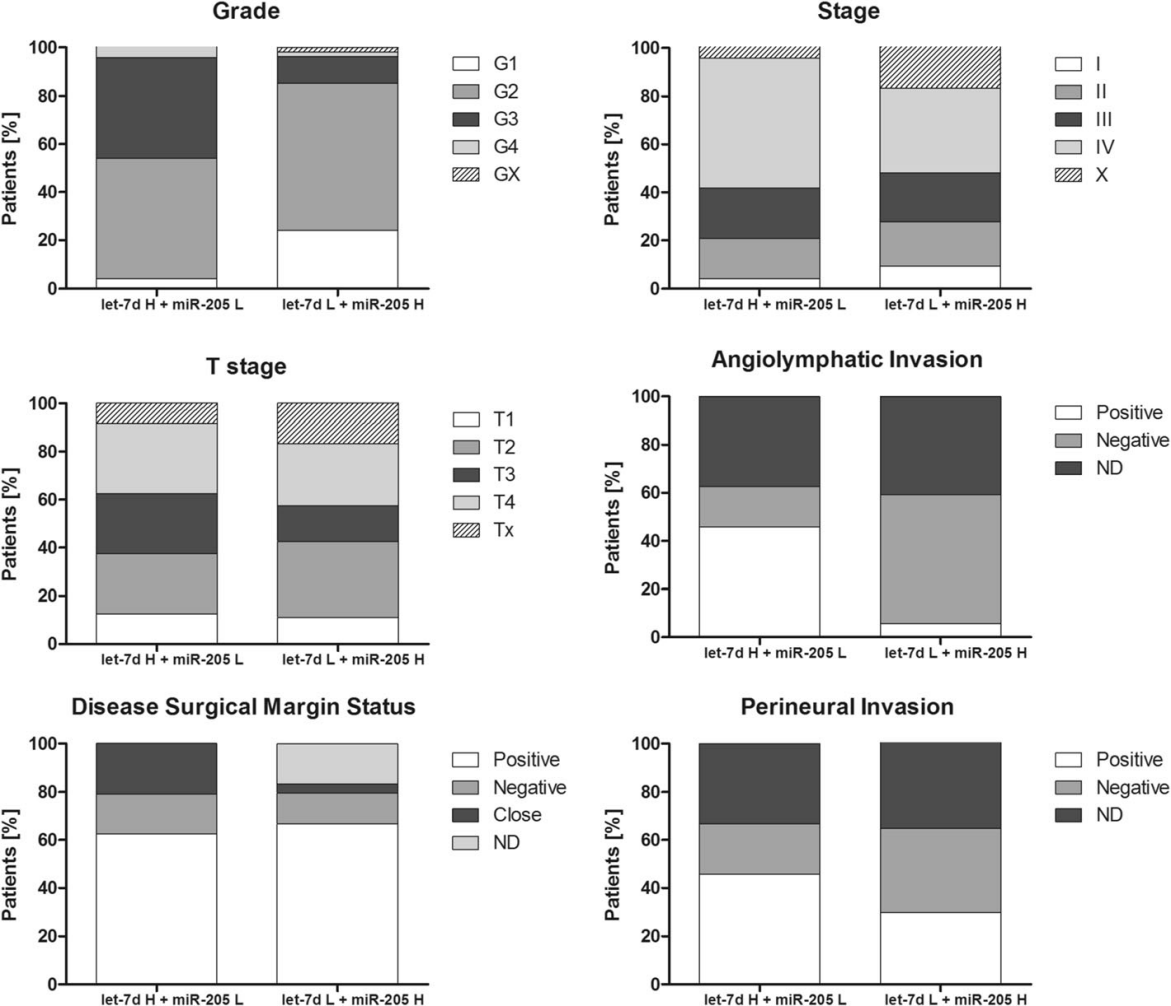
Clinicopathological parameters in HNSCC patient groups with high let-7d and low miR-205 levels (let-7d H + miR-205 L) and low let-7d and high miR-205 levels (let-7d L + miR-205 H); GX, X, and Tx represent indefinite parameter; ND represents no data

The clinicopathological parameters were analyzed in the low let-7d and high miR-205 expression level group and the high let-7d and low miR-205 expression level group. The low let-7d and high miR-205 expression level group had lower percentages of more advanced cancers compared to the high let-7d and low miR-205 expression level group as indicated by the following parameters: cancer grade (G1 + G2: 85.18% vs. 54.17%; G3: 11.11% vs. 41.67%, respectively), cancer stage (I + II: 27.78% vs. 20.84%; III + IV: 37.56% vs. 75.00%, respectively), T-stage (T1 + T2: 42.59% vs. 37.50%; T3 + T4: 42.60% vs. 54.17%, respectively), angiolymphatic invasion (positive: 5.56% vs. 45.83%; negative: 53.70% vs. 16.67%, respectively), disease status of surgical margin (positive: 66.67% vs. 62.50%; negative: 12.96% vs. 16.67%, respectively), and perineural invasion (positive: 29.63% vs. 45.83%; negative: 35.19% vs. 20.83%, respectively). Graphical presentation of the results is shown in Fig. 3.

### Patients with low let-7d and high miR-205 expression level have a distinct tumor phenotype

**Fig. 4:**
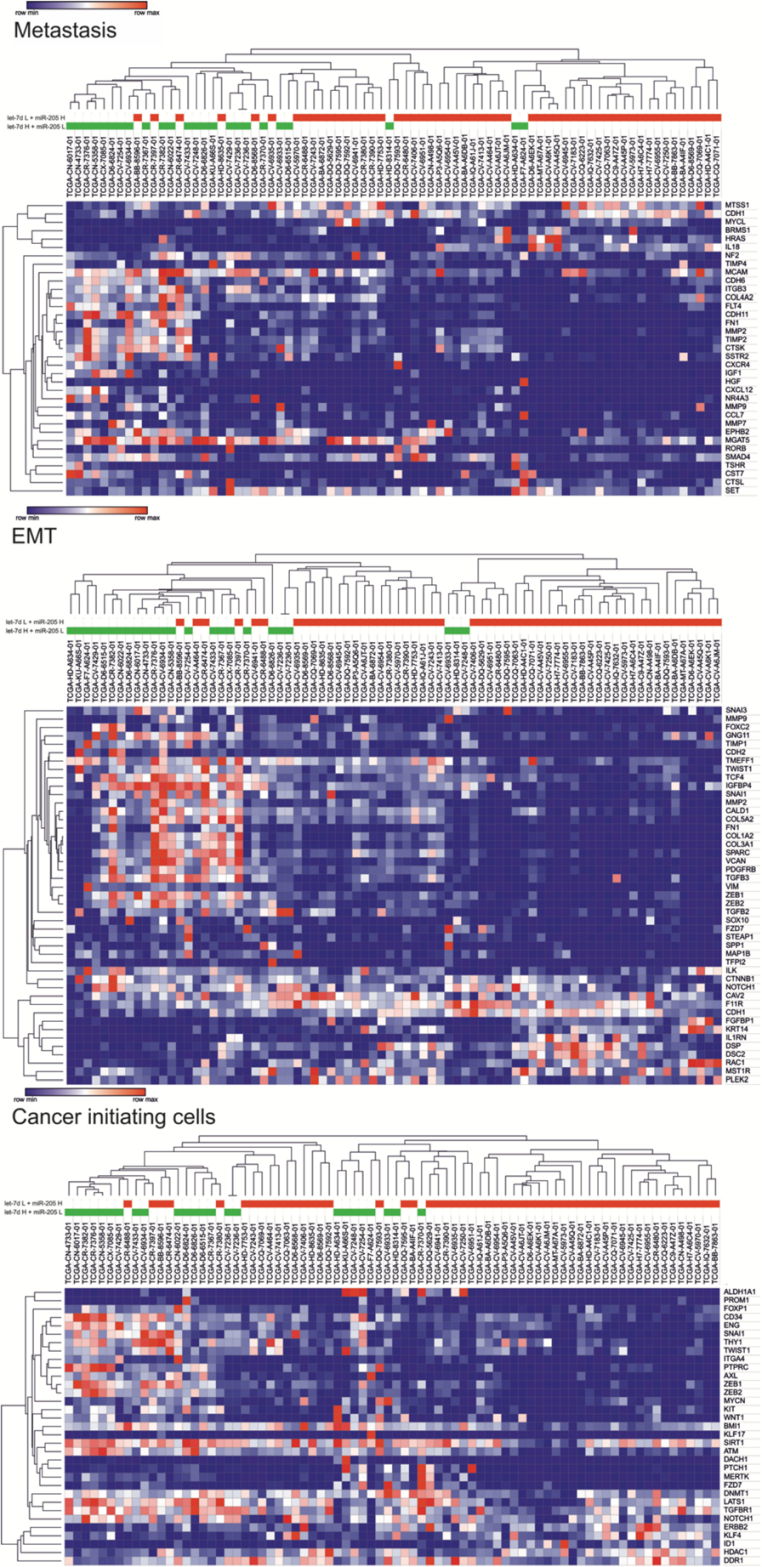
Heat map and clustering of significantly changed (p < 0.05) expression levels of genes related to metastasis, EMT and cancer-initiating cells (mRNA expression z-Scores, RNA Seq V2 RSEM; z = 2). Patients belong to the let-7d high and miR-205 low expression group (let-7d H + miR-205 L) marked as green and patients belong to the let-7d low and miR-205 high expression group (let-7d L + miR-205 H) marked as red

**Fig. 5:**
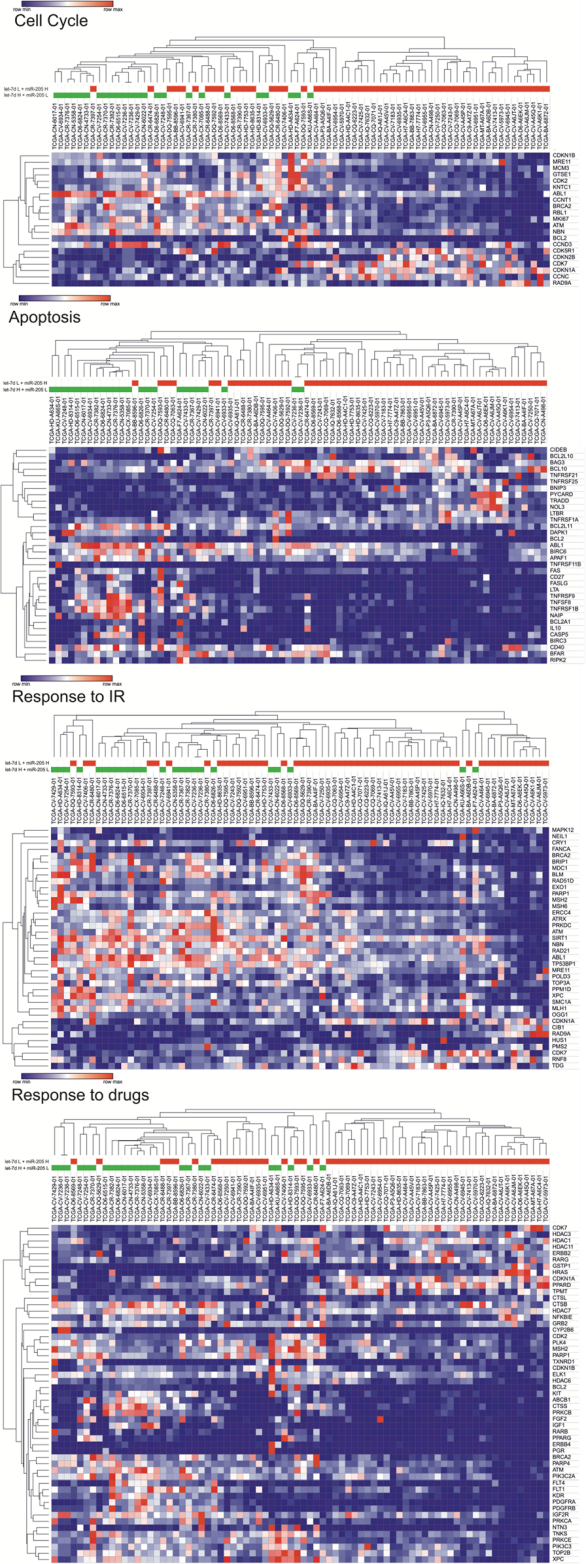
Heat map and clustering of significantly changed (*p* < 0.05) expression levels of genes related to cell cycle, apoptosis, irradiation response and drug response (mRNA expression z-Scores, RNA Seq V2 RSEM; z = 2). Patients belong to the let-7d high and miR-205 low expression group (let-7d H + miR-205 L) marked as green and patients belong to the let-7d low and miR-205 high expression group (let-7d L + miR-205 H) marked as red

For the high let-7d and low miR-205 expression level group (let-7d H + miR-205 L) and the low let-7d and high miR-205 expression level group (let-7d L + miR-205 H), the expression levels of genes related to metastasis, EMT, cancer-initiating cells, cell cycle, apoptosis, irradiation response and drug response were examined and compared between these two groups. A distinct expression pattern of these genes was observed for each group. The let-7d L + miR-205 H group showed the following statistically significant (*p* < 0.05) changes compared to the let-7d H + miR-205 L group: 29 down-regulated and 6 upregulated genes related to metastasis; 31 down-regulated and upregulated 8 genes related to EMT; 28 down-regulated and 5 upregulated genes related to cancer-initiating cells; 17 down-regulated and 4 upregulated genes related to cell cycle; 24 down-regulated and 10 upregulated genes related to apoptosis; 32 down-regulated and 5 upregulated genes related to irradiation response; and 44 down-regulated and 9 upregulated genes related to drug response (Figs. 4 and 5).

**Fig. 6:**
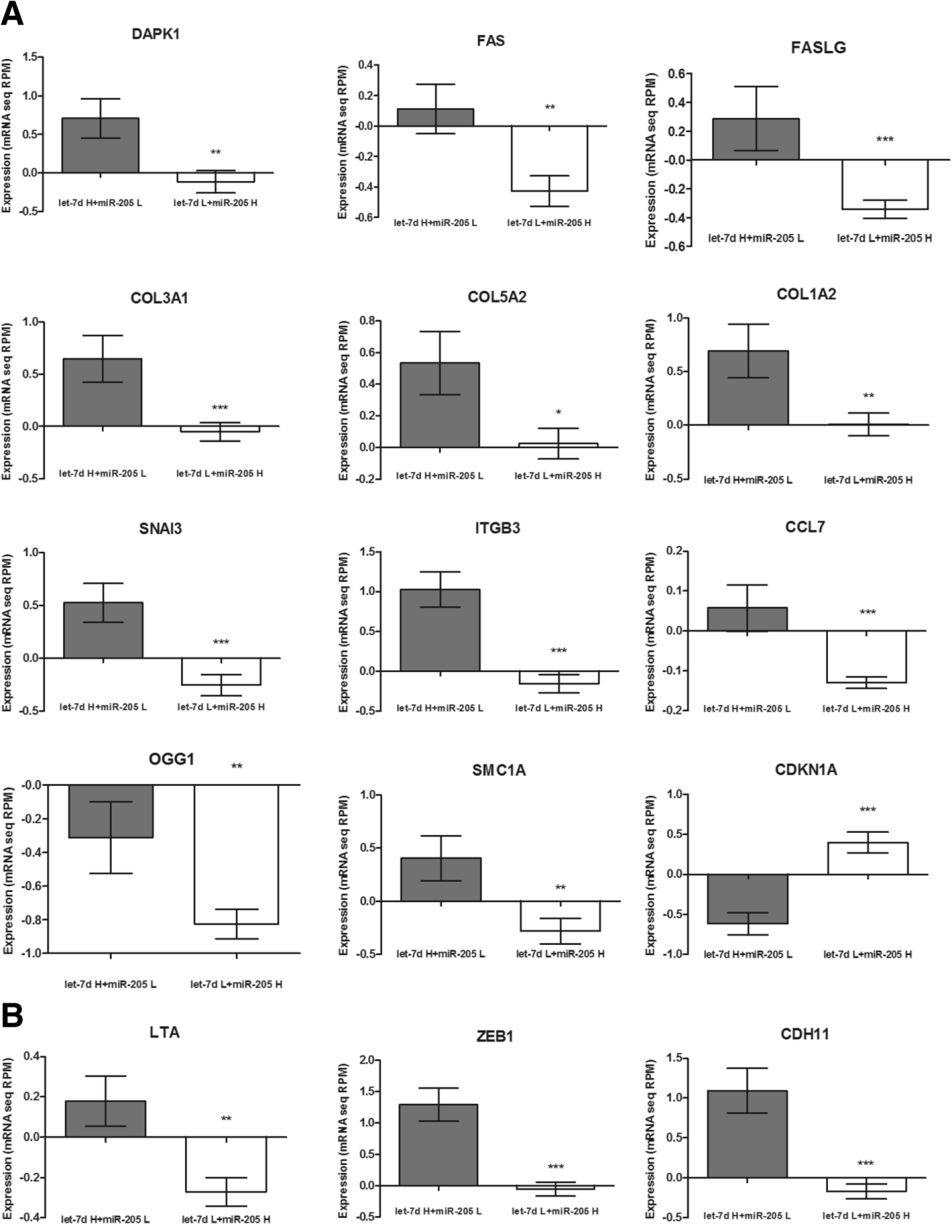
Expression of predicted targets for let-7d (A) and miR-205 (B) in HNSCC patient groups with high let-7d and low miR-205 expression levels (let-7d H + miR-205 L) and low let-7d and high miR-205 expression levels (let-7d L + miR-205 H); unpaired T-test; mean of mRNA expression z-Scores (RNA Seq V2 RSEM; z = 2) with SEM; * *p* < 0.05; ** *p* < 0.01; *** *p* < 0.001

Next, target genes for let-7d and miR-205 were selected from the studied processes using the miRDB database. Twelve targets, including 11 upregulated targets, were identified for let-7d (patients with high let-7d vs. low let-7d) in the set of genes related to metastasis, EMT, cancer-initiating cells, cell cycle, apoptosis, irradiation response and drug response. The following targets were upregulated: DAPK1 (0.7075 ± 0.2553 vs. -0.1161 ± 0.1453; *p* = 0.0039) and FAS (0.1111 ± 0.1606 vs. -0.4269 ± 0.1002; *p* = 0.0046), which are related to negative regulation of apoptosis; FASLG (0.2874 ± 0.2214 vs. -0.3418 ± 0.06343; *p* = 0.0006), which is involved in positive apoptosis regulation; COL3A1 (0.6484 ± 0.2238 vs. -0.04903 ± 0.08634; *p* = 0.0007), COL5A2 (0.5338 ± 0.1996 vs. 0.02531 ± 0.09580; *p* = 0.0112), COL1A2 (0.6935 ± 0.2510 vs. 0.006604 ± 0.1066; p = 0.0039) and SNAI3 (0.5260 ± 0.1833 vs. -0.2556 ± 0.1024; *p* = 0.0002), which are related to EMT; transmembrane receptor ITGB3 (1.027 ± 0.2215 vs. -0.1574 ± 0.1167; *p* < 0.0001) and CCL7 cytokine, which are related to cell growth and proliferation (0.05701 ± 0.05865 vs. -0.1297 ± 0.01475; *p* < 0.0001); and OGG1 (− 0.3112 ± 0.2123 vs. -0.8249 ± 0.08819; *p* = 0.0096) and SMC1A (0.4036 ± 0.2117 vs. -0.2798 ± 0.1203; *p* = 0.0038), which are related to DNA damage and repair. Only one let-7d target was down-regulated, namely CDKN1A (− 0.6176 ± 0.1373 vs. 0.3994 ± 0.1293; p < 0.0001), which is related to cell cycle arrest (Fig. 6a). The following three molecular targets were identified for miR-205 (low miR-205 vs. high miR-205 expression): LTA (0.1778 ± 0.1251 vs. -0.2701 ± 0.07117; *p* = 0.0014), which is a positive regulator of apoptosis; ZEB1 (1.294 ± 0.2672 vs. -0.05386 ± 0.1134; *p* < 0.0001), which is a regulator of cell migration, metastasis, growth and proliferation; and CDH11 (1.092 ± 0.2816 vs. -0.1691 ± 0.09423; *p* < 0.0001), which is related to cell adhesion (Fig. 6b).

## Discussion

Head and neck squamous cell carcinoma (HNSCC) is a group of poor outcome cancers due to late diagnosis, aggressiveness and high capability of metastasis to lymph nodes and distant sites [1, 2, 23]. HNSCC treatment is based on surgery, chemotherapy and radiotherapy, but these treatment strategies are still not personalized and not matched to molecular features of every patient’s cancer [2, 5]. Moreover, HNSCCs contain a small population of tumorigenic and therapy-resistant cells called cancer-initiating cells (CICs). The epithelial-to-mesenchymal (EMT) process is one of the factors responsible for maintenance of this cell phenotype as well as metastasis or poor response to irradiation, chemotherapeutic drugs or other agents [24]. This cell phenotype and behavior are, in part, controlled by miRNAs [3].

Childs et al. proposed that low level miR-205 is associated with loco-regional recurrence independent of disease severity at diagnosis and treatment. Moreover, these researchers postulated that combination of low levels of let-7d and miR-205 expression is associated with poor survival and that these two miRNAs can be used as prognostic markers of HNSCC [7]. However, these results have not been validated using different/larger cohort of patients and the molecular basis of this observation has not been elucidated. The present study used TCGA data and analyzed 307 patients with HNSCC to investigate the diagnostic utility of let-7d and miR-205. The present results contradicted those reported by Childs and colleagues. In the present study, patients with low let-7d and high miR-205 levels had significantly better survival than patients with opposite phenotype - high let-7d and low miR-205 levels. Due to differences in survival between these groups, it was checked whether they differ in terms of other clinical features. We observed, that in the low let-7d and high miR-205 expression level group, there was a lower percentage of more advanced cancers.

It should be noted, that unexpectedly, the small group of patients with low let-7d and miR-205 expression levels was characterized by the best survival. However, these patients comprised 4% of all analyzed cases and due to the small number of cases, this group was not further analyzed by us.

Our analysis confirmed that both let-7d and miR-205 were frequently upregulated or remained unchanged in HNSCC. Other studies have also indicated that let-7d and miR-205 are upregulated in HNSCC [6] and in other cancers [4, 18, 19].

We observed, that patients with higher levels of let-7d and lower levels of miR-205 had worse outcome and more advanced cancers than the group with low let-7d and high miR-205 expression levels.

More advanced tumors (higher N-stage and grade) that generally metastasize to blood and lymph vessels had higher levels of let-7d. Similarly, Hilly et al. found that higher expression of let-7d (and other members of the let-7 family) is associated with more aggressive oral tongue carcinoma in young patients [25]. Based on an in vitro model, Di Fiore et al. demonstrated that let-7d also acts as an oncogene [10]. Our previous study using a FaDu cell line (squamous cell carcinoma of the hypopharynx) with upregulated let-7d expression showed that higher levels of let-7d causes more irradiation-resistant cells but not chemotherapeutic drug-resistant cells [9]. In contrast, down-regulation of let-7d promotes EMT and higher self-renewal of cancer-initiating cells as well as influences the chemoresistant property of oral cancer cells [13]. However, the present results based on TCGA data indicated that let-7d can act as oncogene.

MiR-205, the second analyzed microRNA may function as a tumor suppressor or an oncogene [18, 19]. miR-205 is an epithelial marker [16, 17], and it is a well known modulator of the EMT process [15]. Jamali et al. performed a meta-analysis and showed that down-regulation of miR-205 is associated with poor prognosis in HNSCC [26]. Our results demonstrated that high expression of miR-205 was related to less aggressive tumors, less metastasis to blood, less metastasis to lymph vessels and lower ability of perineural invasion. The present findings also indicated that patients with high miR-205 expression had no dissection of neck lymph nodes.

Clinical and pathological parameters indicate specific, different phenotypes between these groups. To check this, we decided to analyze some important processes such as: cell cycle, apoptosis, EMT, cancer-initiating cells, metastasis, irradiation response and drug response between these groups. Next, we checked the role of let-7d and miR-205 in these processes. We observed differences in the expression of genes associated with the analyzed processes between patients with higher levels of let-7d and lower levels of miR-205 than the group with low let-7d and high miR-205 expression levels.

In the group of patients with low let-7d and high miR-205, the analysis of predicted targets for let-7d showed that the predicted targets, with the exception of CDKN1A (p21), were down-regulated and that these genes were related to apoptosis, EMT, cell cycle, cell proliferation and DNA damage and repair. Llanos et al. indicated that HNSCC patients with p21- and phosphoS6-positive tumors present a better disease-specific survival [27]. Moreover, patients with p21-positive tumors have better overall survival than those with p21-negative tumors [28]. However, immunohistochemical staining of p21 and Ki-67 has shown that coexpression of p21/Ki-67 is a strong negative prognostic factor in HNSCC and may be important in patients treated by primary radiotherapy [29]. p21 can function as a suppressor or oncogene, and it is an example of an antagonistic duality molecule, in which function is determined by particular cellular context [30]. In contrast, the present results suggested that let-7d influences a specific phenotype of HNSCC through direct regulation of CDKN1A and results in better patient survival.

In the analyzed processes we observed three miR-205 targets - LTA, ZEB1 and CDH11, which were down-regulated in the group of patients with low let-7d and high miR-205 compared to the group of patients with high let-7d and low miR-205 expression levels. The LTA gene-encoded tumor necrosis factor-beta (TNF-β), which is a member of the large family of cytokines, is down-regulated in HNSCC compared to normal samples, but TGF receptors RI and RII are upregulated in HNSCC [31]. Buhrmann et al. reported that TNF-β increases the capacity of cell survival and invasion as well as positively influences the EMT process and maintenance of the cancer-initiating cell-like phenotype in colon cancer. Moreover, TNF-β promotes the chemoresistance of colon cancer cells to 5-FU [32].

Based on the HNSCC samples, the high expression of ZEB1 and ZEB2 was associated with advanced and metastatic cancer, and high levels of ZEB1 and ZEB2 predicted poor outcome. ZEB1 and ZEB2 are key modulators of cancer-initiating cell properties in HNSCC, EMT process, metastasis and cisplatin resistance [33]. Kurihara et al. showed that BMI1 and ZEB1 are important factors for promotion of EMT and invasion of tongue cancer. Expression of ZEB1 positively correlates with vimentin, but no significant correlation between ZEB1 and E-cadherin has been identified [34]. The present analysis indicated that the group of patients with better survival had lower levels of cadherin-11 (CDH11). Ma et al. showed that the expression of CDH6, CDH11 and CD44 is upregulated in cancer tissue compared to normal mucosa and is highly increased in OSCC patients with lymph node metastasis. Moreover, OSCC patients with high coexpression of CDH6, CDH11 and CD44 exhibit lower disease-specific survival time, and these molecules may be used as biomarkers of metastases and prognosis [35]. In addition, downregulation of CDH11 promotes proliferation and enhances invasion, and CDH11 has tumor suppressor function. However, overexpression of CDH11 is cancer-specific [36].

## Conclusion

In conclusion, the present study clearly shows that patients with low let-7d and high miR-205 expression levels had less advanced cancers and better survival compared to patients with high let-7d and low miR-205 expression levels. These results contrasted the model presented by Childs and colleagues [7], but the present observations were based on a larger group of patients and were consistent with other studies that describe let-7d as an oncogene [9, 10]. Down-regulation of let-7d and overexpression of miR-205 create a unique cell phenotype with different behavior that influences patient survival compared to cells with upregulated let-7d and down-regulated miR-205. Thus, let-7d and miR-205 are responsible for the observed phenomenon by direct regulation of CDKN1A (let-7d) and by regulation of LTA, ZEB1 and CDH11 (miR-205). However, other mechanisms may better explain the observed results. In conclusion, let-7d and miR-205 are good candidates for new biomarkers in the personalization of HNSCC treatment.

## Abbreviations

5-FU: 5-fluorouracil
AUC: Area under a ROC curve
BMI1: Polycomb ring finger 1
CCL7: C-C motif chemokine ligand 7
CD44: Cluster of differentiation 44
CDH11: Cadherin 11
CDH6: Cadherin 6
CDKN1A: Cyclin dependent kinase inhibitor 1A
CICs: Cancer-initiating cells
COL1A2: Collagen type I alpha 2 chain
COL3A1: Collagen type III alpha 1 chain
COL5A2: Collagen type V alpha 2 chain
DAPK1: Death-associated protein kinase 1
DFS: Disease free-survival
EMT: Epithelial–mesenchymal transition
FAS: Fas cell surface death receptor
FASLG: Fas ligand
HNSCC: Head and neck squamous cell carcinoma
HPV: Uman papilloma virus
ITGB3: Integrin subunit beta 3
Ki-67 (mKi-67): Antigen identified by monoclonal antibody Ki 67
LTA: Lymphotoxin alpha
OGG1: 8-oxoguanine DNA glycosylase
OS: Overall survival
ROC: receiver operating characterisric curve
SMC1A: Structural maintenance of chromosomes 1A
SNAI3: Snail family zinc finger 3
TCGA: The Cancer Genome Atlas
TNF-β: Tumor necrosis factor-beta
UTR: Untranslated region
ZEB1: Zinc finger E-box binding homeobox 1
ZEB2: Zinc finger E-box binding homeobox 2
